# Weightbearing assessment to guide nonoperative treatment of lateral malleolar fractures: the paradigm change

**DOI:** 10.2340/17453674.2025.44878

**Published:** 2025-10-16

**Authors:** Emilia Möller RYDBERG, Kristian PILSKOG, Harri PAKARINEN, Per Henrik RANDSBORG, Charles L SALTZMAN, Marius MOLUND

**Affiliations:** 1 Institute of Clinical Sciences, Sahlgrenska Academy, University of Gothenburg, Gothenburg, Sweden; 2 Department of Orthopaedics, Sahlgrenska University Hospital, Gothenburg, Region Västra Götaland, Sweden; 3 Center for Digital Health, Sahlgrenska University Hospital, Gothenburg, Region Västra Götaland, Sweden; 4 Department of Orthopedic Surgery, Haukeland University Hospital, Bergen, Norway; 5 Department of Orthopaedics and Traumatology, Oulu University Hospital, Oulu, Finland; 6 Department of Orthopaedic Surgery, Akershus University Hospital, Lørenskog, Norway; 7 Institute of Clinical Medicine, Campus Ahus, University of Oslo, Norway; 8 Department of Orthopaedic Surgery, University of Utah, USA; 9 Department of Orthopedic surgery, Østfold Hospital, Grålum, Norway

## Abstract

The goal of this *Acta Orthopaedica* educational article is to provide an update on how to evaluate lateral malleolar ankle fractures at the level of the syndesmosis and to guide clinicians in selecting the most appropriate treatment method. We aim to clarify the indications for non-surgical treatment and to provide clinicians with an evidence-based approach to decision-making in these frequently encountered injuries. The authors introduce the concept of “congruent on weightbearing” in contrast to the historical thinking of ankle fractures as stable or unstable. We further elaborate on how this thinking should be the basis in the decision-making regarding treatment method to safely differentiate fractures that will heal uneventfully without surgical intervention from those that need internal reduction and stabilization.

As long as crucial parts of the deltoid ligament are intact, lateral malleolar ankle fractures at the level of the syndesmosis maintain, or regain, joint congruency under weightbearing. Ankle fractures that stay congruent under weightbearing often heal uneventfully and can be safely treated without surgery. Furthermore, research has shown that early weightbearing and short immobilization periods are beneficial for patient recovery without an increase in complication rates.

Take-home message

- For lateral malleolar fractures at the level of the syndesmosis, weightbearing radiographs taken 4–10 days post-injury should be the decision basis regarding treatment method.
- Fractures that regain or maintain joint congruency under weightbearing are to be treated non-surgically in a cast or functional brace for 3–6 weeks.
- Full weightbearing is advocated.

The management of lateral malleolar ankle fractures at the level of the syndesmosis is undergoing an important transformation—one that challenges long-standing surgical traditions. For decades, much attention has rightly focused on the treatment of unstable ankle fractures. In contrast, careful diagnosis and management of stable fractures have often received less scrutiny. That paradigm is beginning to shift.

Historically, the stability of the ankle mortise was considered primarily dependent on the reduction of the distal fibular fracture. Publications from the 1970s led orthopedic practice to focus on treating lateral malleolus fractures with anatomical reduction, with a 2-mm lateral diastasis of the fibular fracture used as a cut-off for surgical treatment. During the past 2 decades, the nuances of the associated ligamentous injuries that occur with lateral malleolar fractures at the level of the syndesmosis have been investigated extensively. A general focus of this work has been to develop a better way to understand which of these fractures are stable or unstable.

While there is broad consensus that markedly displaced fractures require surgical fixation, emerging evidence shows that even some displaced fractures may maintain joint congruity under weightbearing and can safely be treated non-surgically. Building on these insights, recent anatomical, biomechanical, and clinical studies have accelerated a reassessment of how we define stability and how we should best treat these injuries [1-4].

Nonoperative management was historically reserved for frail patients or for clearly stable, isolated lateral malleolar fractures. But as our understanding of fracture mechanics deepens, so too does our ability to identify patients who may safely avoid surgery as this is not without risk. Wound complications, hardware irritation, and the need for secondary procedures are well-documented concerns [5,6]. Additionally, the inherent risks of anesthesia and postoperative recovery must be weighed carefully. As a result, surgery should be reserved for patients with fractures that cannot maintain joint congruency through the healing process. When joint congruency is preserved without intervention, nonoperative treatment is not only appropriate but preferable. Recent biomechanical and clinical studies have shown that as long as crucial parts of the deltoid ligament are intact, lateral malleolar ankle fractures that maintain, or regain, joint congruency under weightbearing are safe to treat non-surgically, reducing the risks involved in unnecessary surgical procedures [1,4,7].

We aim to clarify the indications for non-surgical treatment and to provide clinicians with an evidence-based approach to decision-making in these frequently encountered injuries.

## Anatomy

**Figure 1:**
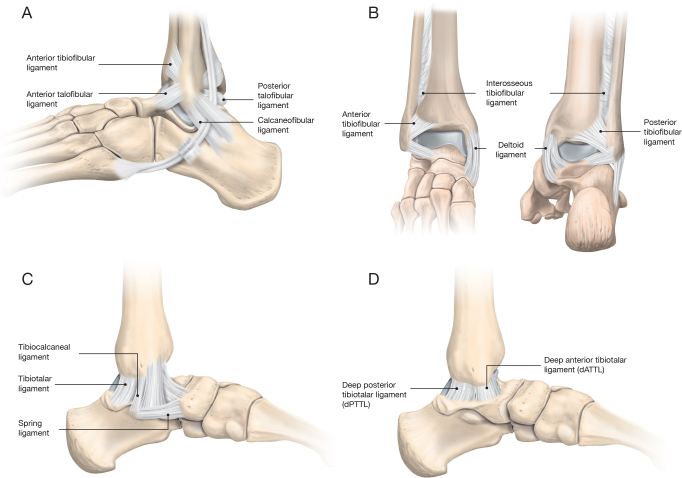
Ligaments surrounding the ankle joint. (A) Ligaments on the lateral side of the ankle. (B) Ligamentous structures on the anterior and posterior aspects of the ankle joint. (C) Superficial components of the deltoid ligament. (D) Deep components of the deltoid ligament.

Stabilization of the bony structures of the ankle is dependent on the lateral ligaments (anterior tibiofibular ligament, anterior talofibular ligament, calcaneofibular ligament, and posterior talofibular ligament) (Figure 1A), the deltoid ligaments on the medial side of the ankle (Figure 1C and D), the posterior tibiofibular ligament, the anterior tibiofibular ligament, and the interosseous tibiofibular ligament (Figure 1B) [8]. The deltoid ligament has 5 components grouped into superficial and deep layers (Figure 1C and D) [7,9]. The superficial layer consists of the tibiotalar, the spring, and the tibiocalcaneal ligaments (Figure 1C). The deep deltoid ligament has anterior (deep anterior tibiotalar ligament [dATTL]) and posterior (deep posterior tibiotalar ligament [dPTTL]) bands (Figure 1D).

The lateral ligaments reduce rotation and inversion of the ankle joint and limit varus stress. The tibiofibular syndesmosis limits widening and motion in the inferior tibiofibular joint [10]. Medially the deltoid ligament resists the joint’s external rotation, eversion, and valgus stresses.

## Classification

The 3 most widely used classification schemes for ankle fractures are those of Weber, Lauge-Hansen, and the system by AO/OTA (Arbeitsgemeinschaft für Osteosynthesefragen/ Orthopaedic Trauma Association) [11-15].

### Weber

The Weber system focuses only on the fibular side of ankle fractures, solely describing the anatomical location of the fracture. At the time of its development, there was a strong appreciation for the importance of restoring fibular length after ankle fracture, but a paucity of interest in medial-sided injuries. Notably, Weber’s classification overlooked the medial side, limiting its usefulness in determining appropriate treatment for the most common ankle fracture: an isolated lateral malleolar fracture at the level of the syndesmosis, termed Weber B.

### Lauge-Hansen

Lauge-Hansen’s classification system employs a 2-part nomenclature, in which the first word denotes the foot’s position at the time of injury, and the second word indicates the direction of the deforming force. Paradoxically, Lauge-Hansen’s cadaver-based work, which was originally intended to help with closed treatment, gives a much better understanding of the pathoanatomy because it describes the temporal order and magnitude of peri-ankle tissue injuries, including the medial side of the joint. The Lauge-Hansen system illuminates the relevant pathoanatomy and establishes a framework to think about how to optimize treatment for Weber B fractures that correspond to Lauge-Hansen’s supination–external rotation (SER) fracture pattern. According to Lauge-Hansen this injury occurs in a foot that is supinated at the time of injury when an external rotational force is applied to the ankle, progressively stressing and injuring some or all specific structures in a 4-stage sequence from lateral to medial.

### AO/OTA

The AO/OTA classification system can be seen as a further development of the Weber classification, combined with the understanding of the injury mechanism by Lauge-Hansen. According to the AO/OTA classification system, B-fractures are subclassified into 1, 2, or 3 depending on the number of involved malleoli and then further sub-grouped according to dislocation, comminution, or presence of a deltoid ligament injury. The AO/OTA equivalent to the SER II fractures is the B1 and the SER IV fracture is the B2.1.

Regardless of classification scheme, on initial non-weightbearing images, it is challenging to distinguish the SER IIs from most IIIs and IVs, or B1s from B2.1, unless there is evidence of bony avulsions from the involved relevant structures or major incongruence in the ankle joint. This limits the usefulness of all these classifications as in many cases they cannot guide choice of treatment in the most common ankle fractures.

## Biomechanics and stability

Historically, the stability of the ankle mortise was thought to rely primarily on the proper reduction of the distal fibular fracture [16]. In a biomechanical study from 1976, Ramsey and Hamilton found that 1 mm lateralization of the talus reduced tibiotalar contact by 42% [16]. Yablon published a study on 51 patients with bimalleolar fractures, claiming that only anatomical reduction of the fibular fracture correctly placed the talus under the tibia [17]. They concluded that the talus follows the distal fibular fragment. These publications changed orthopedic practice to focus on treating lateral malleolus fractures with anatomical reduction.

Already in the 1980s, Bauer et al. had found that the degree of displacement of the fibular fracture does not predict the displacement of the talus under axial load of the ankle [18,19]. Studies reported good long-term outcomes in fibular fractures with up to 5 mm dislocation [18,20]. Recent clinical studies have demonstrated excellent clinical outcomes in Weber B ankle fractures with no concomitant sign of injury to the medial side when treated with early weightbearing and no cast immobilization [21,22]. This further emphasizes that the fibular fracture alone does not introduce significant instability to the ankle.

In the mid-1990s, Michelson et al. published several studies on ankle fractures [23-25]. Their work suggests that the main stabilizing structure of the ankle is not the lateral malleolus, but rather the medial side of the ankle as the talus shifted laterally only after complete sectioning of the deltoid ligaments [25].

To better distinguish stable from unstable fractures, Gougouglias and Sakellariou proposed subclassifying SER IV ankle fractures into SER IVa and SER IVb based on their understanding of the deep deltoid anatomy [26]. They recognized the probable differential function of the 2 components of the deep deltoid: (a) the inconstant and smaller deep anterior tibiotalar ligament (dATTL) and (b) the constant, larger, and stronger deep posterior tibiotalar ligament (dPTTL). Importantly, they noted that the dPTTL tightens when the foot is plantigrade or dorsiflexed and loosens when the foot is plantarflexed. Based on this, Gougouglias and Sakellariou divided SER IV equivalent medial ligament injuries into 2 types:

- SER IVa: lateral malleolar fracture with a ruptured superficial deltoid and/or dATTL but an intact dPTTL.
- SER-IVb: lateral malleolar fractures with a complete rupture of the superficial and deep components (both dATTL and dPTTL).

They postulated that when the SER IVb type full deltoid ligament injury occurs, the medial clear space will open in all positions of the foot, including on weightbearing radiographs. Most cadaver-based studies show that they were directionally correct [4,27].

In a recent biomechanical study, the superficial deltoid ligament and the dATTL were stretched and came under tension during supination and plantar flexion [7], while the dPTTL did not come under tension during this movement. The dPTTL is put under tension during dorsiflexion of the ankle. Thus, dorsiflexion during weightbearing of the ankle tensions the deep deltoid ligament, which pulls the talus into an anatomical position centered under the tibia. Dalen et al. examined the effect of successive sectioning of the deltoid ligament bands [4]. They found a substantial change in instability between partial and complete deltoid injuries. Simulated Weber B fractures with injuries to the superficial and dATTL with an intact dPTTL were unstable in plantarflexion and external rotation—the assumed mechanism of injury in Weber B fractures. However, these injuries were stable in dorsiflexion and external rotation. Complete deltoid injuries were unstable in all directions and positions of the ankle (plantarflexion, neutral, dorsiflexion, internal/external rotation).

This new understanding of the biomechanics of the deltoid ligament challenges the old assumptions that all ankle fractures with signs of deltoid ligament injury are unstable. It explains that a large proportion of ankle fractures have only partial deltoid ligament ruptures, as the dPTTL is protected through the trauma mechanism. These ankles may show signs of medial clear space widening on stress testing but are stable under dorsiflexion and weightbearing.

## Assessment and management of lateral malleolar fractures

### Evaluation

As the deltoid ligament is suggested as the primary stabilizer of the loaded ankle, clinicians have investigated Weber B ankle fractures for signs of deltoid ligament injury under the assumption that any concomitant sign of deltoid ligament injury would cause the ankle to be unstable. Many have written that the examination should include a careful assessment of the medial side of the ankle joint to assess swelling, tenderness, and ecchymosis. As part of the overall examination, this is perfectly appropriate. Still, not too much value should be given to the findings as studies repeatedly show that the value of those findings with respect to whether medial ankle ligaments are intact is highly unreliable [28-30].

In 2004, a manual external rotation stress test was proposed as a method to distinguish stable from unstable fractures. A few years later, a gravity stress radiograph was advocated [31]. Both rely on measurement of the medial clear space (MCS) on a non-weightbearing mortise ankle radiograph.

Often MCS > 4 mm was used to identify patients deemed likely to be unstable and thus carry a high risk for malunion and the development of post-traumatic degenerative ankle arthritis. However, there is no scientific basis in picking > 4 mm as the cutoff threshold for predicting the long-term outcomes of SER ankle fractures. In addition, these images were not taken while the subjects were bearing weight. The tibiotalar joint’s ability to naturally self-center and the substantial restraint that comes from articular contact and geometry are completely ignored [32-34]. The gravity stress test is found to overestimate the instability of the ankle, and the need for these tests has been diminished due to the development of the weightbearing radiographs [3,23,35].

In 2010, Martin Weber and colleagues reported on the use of “weightbearing radiographs to distinguish stable and unstable, isolated lateral malleolus fracture induced by the SER mechanism” [23]. Based on their criteria for instability, 90% of SER fractures were able to be treated nonoperatively. Hoshino et al. evaluated patients with a known positive radiographic manual stress test with weightbearing radiographs taken 1 week later [35]. 3 patients of 38 studied were considered to possibly have unstable ankle fractures. They were offered surgical treatment. Thus 90% of patients who would have been indicated for surgery based on manual stress testing were ultimately considered to have stable ankle fractures and were treated without surgery [35].

**Figure 2:**
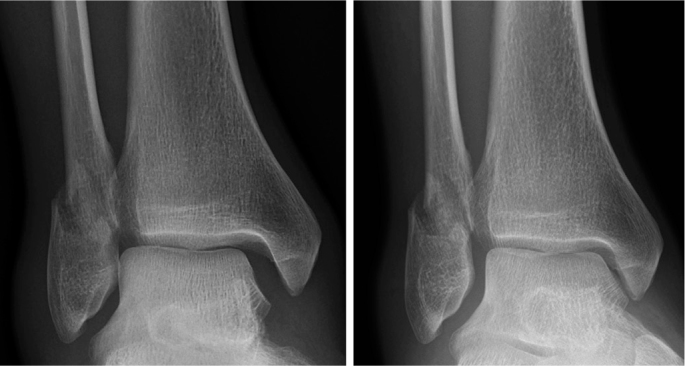
Comparison of non-weightbearing and weightbearing radiographs in a lateral malleolar fracture. Left: Widening of the medial clear space (MCS) on non-weightbearing views. Right: Restoration of ankle congruency with weightbearing.

As already described, several anatomical and biomechanical studies suggest that a proportion of lateral malleolar ankle fractures have a partial deltoid ligament rupture, which could appear incongruent on conventional radiographs but be stable in dorsiflexion of the ankle (Figure 2). Therefore, to better assess the ankle joint stability, the weightbearing test has emerged as the recommended evaluation method [1,3,23, 35-37] .

**Figure 3:**
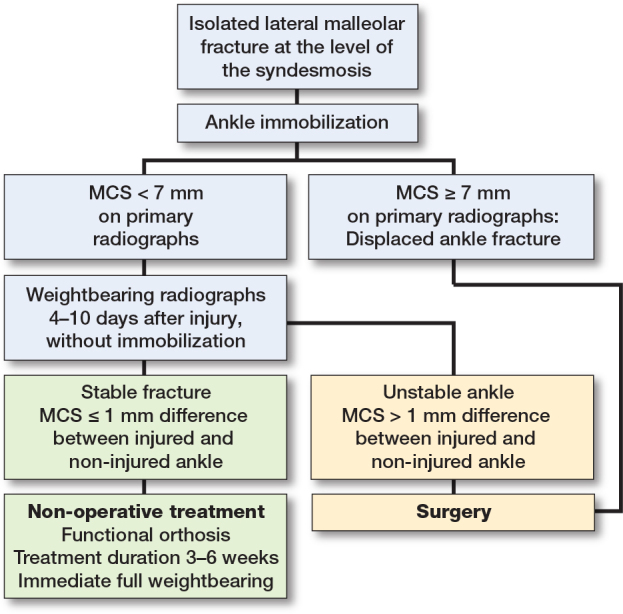
Flowchart outlining the use of weightbearing radiographs to guide treatment of lateral malleolar fractures at the syndesmosis level.

**Figure 4:**
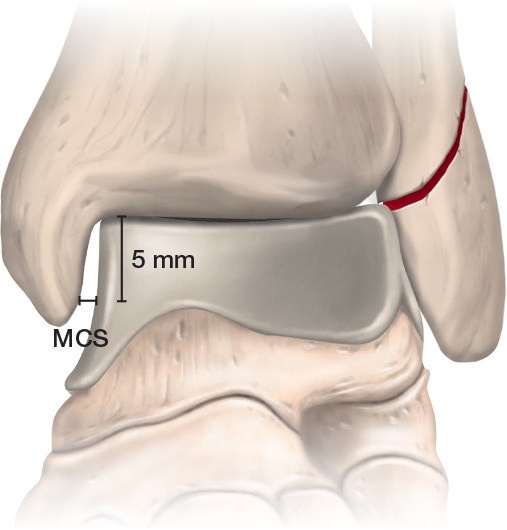
Demonstration of how the medial clear space (MCS) is measured.

**Figure 5:**
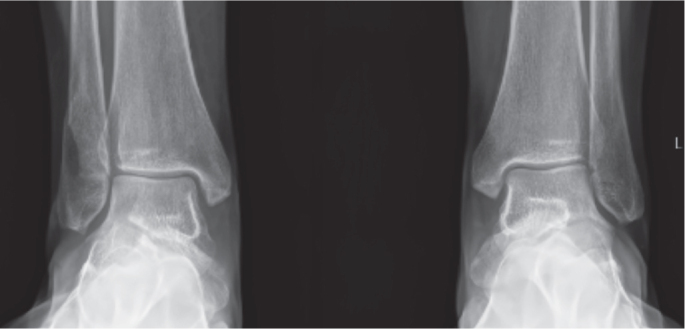
Weightbearing radiographs demonstrating symmetric ankle mortises in the injured and non-injured ankles.

The weightbearing radiographs should be taken when the acute pain has subsided and the patient is able to put equal weight on both feet, but still within the window of opportunity for surgical treatment should the ankle joint not be congruent. The recommended timing for weightbearing radiographs is 4–10 days post-injury. As shown in the flowchart (Figure 3), patients with lateral malleolar fractures at the level of the syndesmosis—who have neither experienced dislocation nor undergone reduction prior to imaging and present with an MCS of up to 7 mm—are eligible for weightbearing radiographs [1,37]. The fracture is temporarily immobilized, and the patient is taken back 4–10 days post-injury for weightbearing radiographs. The cast or splint is removed prior to the weightbearing radiographs being taken. To properly evaluate joint congruency, it is crucial that the patient is able to bear 50% of bodyweight on the injured foot when the radiographs are taken [38]. MCS is measured on the weightbearing radiographs (Figure 4). In most cases the ankle mortise is perfectly reduced on weightbearing radiographs and stability is easily determined. In cases of any doubt the MCS of the injured ankle can be compared with the non-injured ankle (Figure 5). If there is less than 1 mm difference in the MCS between injured and non-injured ankle, nonoperative treatment is continued. This is consistent with studies based on weightbearing computed tomography showing minimal intrasubject variation in ankle rotation and alignment, supporting the contralateral ankle as the best reference for assessing injury-related displacement [39].

## Treatment

Stable ankle fractures have been treated with immobilization in a below-the-knee cast at a 90° angle [40-42] . Recommendations for immobilization periods have varied from 4 to 6 weeks depending on the method used to determine the stability of the fracture [40,41,43-45]. Failures of non-surgical treatment have been associated with inadequate assessment of ankle fracture stability [46].

However, prolonged cast immobilization of the lower limb has revealed several adverse effects, including decreased range of motion, muscle atrophy, diminished strength, and an increased risk of deep vein thrombosis [41,47-49]. Problems associated with cast treatment might be avoided by treatment of stable ankle fractures with simple, more functional measures, such as braces, orthoses [48,50], or strapping [43]. These functional treatment methods are suggested to result in more rapid rehabilitation and better short-term functional outcome when compared with traditional cast immobilization [48]. Multiple studies on the postoperative regime for surgically treated ankle fractures have concluded that full immediate weightbearing and early range of motion improve short-term functional outcomes and lead to earlier return to work and pre-injury activities, without an increase in complications [51-56].

In ankle fracture studies that utilize weightbearing imaging around 4–10 days post-injury to plan treatment, further treatment has been administered either with a cast or a removable ankle orthosis for 3–6 weeks [23,35,37].

## Outcomes

Given the challenges of old studies with many limitations, we focus on relatively recent, prospectively designed investigations to answer key clinical questions. One such study, the Combined Randomised and Observational Study (CROSSBAT) by Mittal et al., evaluated patients aged 18 to 65 with isolated Weber B ankle fractures and minimal talar shift [57]. The study included both a randomized cohort (72 surgical and 69 non-surgical patients) and an observational cohort (19 surgical and 257 non-surgical patients). At 12 months post-injury, the study found no significant advantage of surgical over non-surgical management in terms of ankle function and health-related quality of life. Surgical management was associated with a higher rate of adverse events. A follow-up study tracked approximately half of the randomized cohort at a mean of 7.3 years (minimum 5 years) and found unchanged results—no clinically or statistically significant differences between surgical and nonoperative management, with a higher rate of adverse events in the surgical group (odds ratio 3.7, 95% confidence interval 1.2–11.6, P = 0.04) [58].

Similarly, researchers from Oulu University Hospital in Finland have published multiple studies on the long-term outcomes of nonoperatively treated “stable” ankle fractures [42,59]. Their longest follow-up study assessed 102 of 160 patients at an average of 12 years post-fracture [59]. The authors utilized the stability-based ankle fracture classification introduced by Michelson et al., categorizing fractures simply as stable or unstable [60]. In the original cohort, 75 patients were classified as having stable fractures, including 52 with Weber B fractures. Although the 2021 long-term follow-up did not specify the distribution of Weber A versus Weber B fractures, the study found that no patients with an ankle fracture deemed stable by this classification required surgical treatment at any point during the 12-year follow-up. Furthermore, functional outcomes remained excellent and unchanged between the 2-year follow-up and the 12-year follow-up, reinforcing the durability of nonoperative management for stable ankle fractures over the long term [42,59].

Most recently, Gregersen et al. conducted a prospective, noninferiority study on 149 patients with Weber B ankle fractures classified as stable (SER2, n = 88) or partially unstable (SER4a, n = 61) based on weight-bearing radiographs [22]. Stability was defined by MCS < 5.0 mm and a difference of ≤ 1.0 mm when comparing the uninjured and the weight-bearing radiographs. All patients were treated with functional orthoses and allowed full weight-bearing. At the 2-year follow-up, outcomes based on the Manchester–Oxford Foot and Ankle Questionnaire (MOXFQ) and Olerud–Molander Ankle Score showed no significant differences between the SER2 and SER4a groups. They further reported maintenance of a normal MCS compared with the uninjured side for these patients, indicating that an unstable gravity stress test (SER4a) did not predict worse clinical or radiographic outcomes.

Together, these studies reinforce the growing evidence supporting nonoperative management for stable lateral malleolar ankle fractures, challenging traditional approaches to surgical intervention and prolonged immobilization. While gaps in long-term data persist, emerging research continues to refine treatment strategies for optimizing patient outcomes.

## Special considerations

Patients with multiple comorbidities, particularly those with end-stage complications of diabetes mellitus, severe osteoporosis, smokers, morbid obesity, intractable alcoholism, or cognitive impairment need special consideration and do not fit into the general schema for decision making. It remains incumbent on the treating physician to recognize and consider that these patients may need to be treated differently. Generally, prolonged weightbearing reduction and casting duration, as well as frequent follow-up radiographs and cast changes (to monitor soft tissue conditions), are recommended for these frail patients [61-63].

Although weightbearing radiographs are highly reliable, subtle ligamentous injuries may occasionally evolve or present later in recovery. Continued clinical monitoring remains important, particularly if unexpected pain, instability, or delayed recovery occurs.

To be able to draw conclusions regarding joint congruency and plan for further treatment it is crucial that the patient can put bodyweight on the injured foot when the weightbearing radiographs are taken. In clinical practice, patients are required to bear approximately half of their bodyweight on the injured foot to obtain radiographs that are suitable for assessment. Should the patient not be able to do this, the radiographs cannot be used for the decision-making.

In addition, the accuracy of weightbearing radiograph interpretation also depends on proper imaging technique. Variations in radiographic technique could impact the assessment of ankle stability and should be carefully controlled.

## Conclusion

Lateral malleolar fractures at the level of the syndesmosis with suspected deltoid ligament injury (clinical signs or widening of the MCS) should be examined with weightbearing radiographs to investigate joint congruency as the decision basis for further treatment. As long as crucial parts of the deltoid ligament are intact, lateral malleolar ankle fractures preserve joint congruency under weightbearing and are “stable enough” to be treated non-surgically, avoiding the risks involved in unnecessary surgical procedures. This approach represents a paradigm shift toward evidence-based, functional management, where weightbearing as tolerated in a cast or functional brace should be advocated and immobilization periods kept short to avoid adverse effects.

## Notes

The authors would like to thank Pontus Andersson, Pontus Art Production, for illustrations in Figures 1 and 4.

Handling co-editor: Jeppe Vejlgaard Rasmussen

*Acta* thanks Teppo L N Järvinen and Bjarke Viberg for help with peer review of this manuscript.
